# Traumatic Brain Injury Alters Cerebral Concentrations and Redox States of Coenzymes Q_9_ and Q_10_ in the Rat

**DOI:** 10.3390/antiox12050985

**Published:** 2023-04-23

**Authors:** Giacomo Lazzarino, Renata Mangione, Miriam Wissam Saab, Barbara Tavazzi, Alessandra Pittalà, Stefano Signoretti, Valentina Di Pietro, Giuseppe Lazzarino, Angela Maria Amorini

**Affiliations:** 1 Departmental Faculty of Medicine and Surgery, UniCamillus-Saint Camillus International University of Health and Medical Sciences, Via di Sant’Alessandro 8, 00131 Rome, Italy; barbara.tavazzi@unicamillus.org (B.T.); stefano.signoretti@aslroma2.it (S.S.); 2 Department of Basic Biotechnological Sciences, Intensive and Perioperative Clinics, Catholic University of the Sacred Heart of Rome, Largo F. Vito 1, 00168 Rome, Italy; renata.mangione@unicatt.it; 3 Department of Biomedical and Biotechnological Sciences, Division of Medical Biochemistry, University of Catania, Via S. Sofia 97, 95123 Catania, Italy; miriam.saab@phd.unict.it (M.W.S.); alessandrapittala@gmail.com (A.P.); amorini@unict.it (A.M.A.); 4 Department of Emergency and Urgency, Division of Neurosurgery, S. Eugenio/CTO Hospital, A.S.L. Roma2 Piazzale dell’Umanesimo 10, 00144 Rome, Italy; 5 Neurotrauma and Ophthalmology Research Group, School of Clinical and Experimental Medicine, College of Medical and Dental Sciences, University of Birmingham, Edgbaston, Birmingham B15 2TT, UK; 6 National Institute for Health Research Surgical Reconstruction and Microbiology Research Centre, Queen Elizabeth Hospital, Edgbaston, Birmingham B15 2TH, UK

**Keywords:** coenzyme Q_9_, coenzyme Q_10_, electron transport chain, energy metabolism, HPLC, mitochondrial dysfunction, oxidative stress, ROS, α-tocopherol, traumatic brain injury

## Abstract

To date, there is no information on the effect of TBI on the changes in brain CoQ levels and possible variations in its redox state. In this study, we induced graded TBIs (mild TBI, mTBI and severe TBI, sTBI) in male rats, using the weight-drop closed-head impact acceleration model of trauma. At 7 days post-injury, CoQ_9_, CoQ_10_ and α-tocopherol were measured by HPLC in brain extracts of the injured rats, as well as in those of a group of control sham-operated rats. In the controls, about the 69% of total CoQ was in the form of CoQ_9_ and the oxidized/reduced ratios of CoQ_9_ and CoQ_10_ were, respectively, 1.05 ± 0.07 and 1.42 ± 0.17. No significant changes in these values were observed in rats experiencing mTBI. Conversely, in the brains of sTBI-injured animals, an increase in reduced and a decrease in oxidized CoQ_9_ produced an oxidized/reduced ratio of 0.81 ± 0.1 (*p* < 0.001 compared with both controls and mTBI). A concomitant decrease in both reduced and oxidized CoQ_10_ generated a corresponding oxidized/reduced ratio of 1.38 ± 0.23 (*p* < 0.001 compared with both controls and mTBI). An overall decrease in the concentration of the total CoQ pool was also found in sTBI-injured rats (*p* < 0.001 compared with both controls and mTBI). Concerning α-tocopherol, whilst no differences compared with the controls were found in mTBI animals, a significant decrease was observed in rats experiencing sTBI (*p* < 0.01 compared with both controls and mTBI). Besides suggesting potentially different functions and intracellular distributions of CoQ_9_ and CoQ_10_ in rat brain mitochondria, these results demonstrate, for the first time to the best of knowledge, that sTBI alters the levels and redox states of CoQ_9_ and CoQ_10_, thus adding a new explanation to the mitochondrial impairment affecting ETC, OXPHOS, energy supply and antioxidant defenses following sTBI.

## 1. Introduction

Traumatic brain injury (TBI) occurs any time an external force, directly or indirectly acting to the head, is transferred in part to the nervous tissue initiating a sudden neurometabolic cascade that alters a plethora of biochemical and molecular processes of cerebral cells [1,2,3]. In the clinical setting, there are numerous variables rendering each TBI different from one another, including (i) the type and intensity of the force acting at the time of impact (penetrating, non-penetrating, static, dynamic); (ii) the sub-type of force, if dynamic (rotational, translational); (iii) the timing between the impact and admission to NICUs; (iv) the genotype, phenotype and epigenotype of a TBI patient. Each of these variables has unpredictable consequences on the evolution of the TBI and on patients’ outcome. Therefore, TBI is probably the most complex pathology involving the central nervous system and, notwithstanding its high incidence [4], still requires valid pharmacologic treatments that positively affect TBI patients’ outcome.

However, thanks to experimental studies in laboratory animals, our knowledge on the biochemical and molecular changes characterizing the post-TBI brain has greatly improved in the last few decades. These TBI-associated alterations of nervous cell functions, known as the secondary insult and lasting for days, weeks and months after a TBI, comprise changes in ionic homeostasis [5], an excess release of excitatory neurotransmitters (glutamate, aspartate) [6], an imbalance of glucose metabolism [7], mitochondrial dysfunction [8], an insurgence of oxidative/nitrosative stress [9], the activation of neuroinflammatory processes leading to cellular apoptosis [10,11] and damage to the blood brain barrier (BBB) permeability [12]. Using the closed-head impact acceleration model of graded TBI [13], previous studies from our research group highlighted the differential effects of mild (mTBI) and severe (sTBI) head trauma on energy and glucose dysmetabolism [14,15] and mitochondrial malfunctioning [16,17], evidencing the recovery of biochemical functions following mTBI and the long-lasting metabolic impairment of nervous cells after sTBI. Alterations of the mitochondrial electron transport chain (ETC) coupled to oxidative phosphorylation (OXPHOS) are crucial determinants in the (transient or permanent) post-TBI energy crisis [18,19].

Conzyme Q (CoQ) is the well-known mobile electron transporter which, thanks to its hydrophobicity, is localized within the phospholipid bilayer of various biological membranes [20]. The majority of CoQ is localized intracellularly in the inner mitochondrial membrane [21], where it provides the transfer of electrons from Complexes I and II to Complex III of the ETC. The quinone ring allows CoQ to perform one or two electron transfers, so that CoQ may exist in its fully oxidized and fully reduced forms, as well as in its semiquinone CoQ radical. This last species is principally generated in the so-called Q cycle, occurring within Complex III of the ETC. The vicinity of molecular oxygen may cause the O_2_ reaction with CoQ^−•^ with the formation of superoxide anion and oxidized CoQ (CoQ ox) [21]. In the case of mitochondrial malfunctioning, the frequency of this reaction increases and the leak of electrons at the Complex III site provokes a net increase in O_2_^−•^ subsequently leading to the formation of other reactive oxygen species (ROS), ultimately resulting in cell oxidative stress [22,23]. While in humans CoQ is 99% present as CoQ_10_, where the number of the deponent indicates the length of the repetitive 5-carbon atoms isoprenoid chain, in other animal organisms there are CoQ with shorter hydrophobic chains [20,21] in different proportions. Rats, one of the animal species very frequently used in experimental studies, have two forms of CoQ: CoQ_9_ and CoQ_10_ [24]. Curiously, the cells of all rat tissues but the brain have high concentrations of CoQ_9_ and minimal amounts of CoQ_10_ [25]. Indeed, although rat cerebral cells have higher CoQ_9_ levels (~70% of total CoQ), their CoQ_10_ concentrations (~30% of total CoQ) are about six times higher than those found in non-nervous cells [25]. 

Notwithstanding the relevant biochemical role of CoQ and the well-defined occurrence of mitochondrial dysfunction, to date there no available data showing whether and how TBI affects both the level and the redox state of this fundamental electron transporter. In order to clarify this issue, in the present study, besides determining the cerebral levels of α-tocopherol for its potential connections with CoQ, we measured the concentrations of the reduced and oxidized species of CoQ_9_ and CoQ_10_ in the brains of control rats and in those of animals a week after experiencing a diffuse type of graded TBI (mTBI or sTBI).

## 2. Materials and Methods

### 2.1. Chemicals and Preparation of Reduced CoQ_9_

HPLC-grade acetonitrile (far-UV) and chloroform were obtained from J. T. Baker Inc. (Phillipsburgh, NJ, USA). Oxidized CoQ_9_, oxidized and reduced CoQ_10_ and α-tocopherol were purchased from Sigma-Aldrich (St. Louis, Mo, USA). Since the reduced form of CoQ_9_ is not commercially available, standard solutions of this compound were obtained by the reduction of oxidized CoQ_9_ with known concentration, using NaBH_4_ as the reducing agent and following the procedure described in [26]. The full conversion of oxidized into reduced CoQ_9_ was confirmed by the absence of the oxidized CoQ_9_ peak in HPLC chromatographic runs, performed immediately after completing the reduction reaction. Stock solutions of reduced CoQ_9_ were stable for at least 72 h at 4 °C.

### 2.2. Induction of Graded TBI

The study was approved by the Ethic Committee of the Catholic University of Rome (approval 1F295.52, released on 20 October 2017) and received approval by the Ethical committee of the Italian Ministry of Health (approval No. 78/2018-PR released on 02/05/2018, and approval n° 304/2022-PR released on 22 May 2022). Male Wistar rats (n = 26) of 300–350 g body weight (b.w.) were kept in the animal house under constant temperature and humidity, fed with a standard laboratory diet and water ad libitum. Anesthesia was induced with an intramuscular injection of 35 mg/kg b.w. ketamine and 0.25 mg/kg b.w. midazolam. Graded TBI was induced according to the closed-head impact acceleration model [13] by dropping a 450 g weight from 1 or 2 m heights onto the helmet protected rat head (fixed on the skull with a proper surgical procedure, immediately prior to trauma induction), consequently causing, respectively, mTBI (*n* = 8) or sTBI (*n* = 8). In the case that rats suffered from skull fracture, seizures or nasal bleeding, they were not included in the study (an overall rate of mortality of 8% was recorded). Seven days after head impact, animals were again anesthetized and sacrificed. Control animals (*n* = 8) received the same surgical procedure (used for helmet fixation) and anesthesia administration as the injured rats and were sacrificed 7 days after surgery.

To validate the analytical characteristics of the HPLC separation of reduced and oxidized CoQ_9_ and CoQ_10_, previously validated to quantify α-tocopherol and other fat-soluble compounds [27], a separate group of naïve control animals (*n* = 6) was anesthetized, immediately sacrificed and subjected to craniectomy and brain removal, as described below. The only difference was that each brain was divided into two hemispheres, one of which was processed as described below, whilst the other was spiked with a standard mixture containing either low (*n* = 3) or high (*n* = 3) concentrations of reduced and oxidized CoQ_9_ and CoQ_10_.

### 2.3. Tissue Processing and HPLC Determination of Reduced and Oxidized CoQ_9_ and CoQ_10_

As described in detail elsewhere [6,14,15,16,17], 7 days after surgical procedures (controls) or TBI induction (mTBI and sTBI rats), anesthetized animals underwent an in vivo craniectomy. The brain was exposed and immediately freeze-clamped by aluminum tongue pre-cooled in liquid N_2_ and then immersed in liquid N_2_, with the aim of keeping to the minimum any possible loss of metabolites. The tissue wet weight (w.w.) was quickly recorded and the frozen brain was deproteinized by 90 s homogenization in HPLC-grade CH_3_CN (acetonitrile), using an Ultra-Turrax homogenizer (Janke and Kunkel, Staufen, Germany) set at the maximal speed (24,000 rpm/min). The solid (tissue) to liquid (CH_3_CN) ratio was calculated on the basis of the tissue wet weight, in order to obtain a 1:4.5 (*w*/*v*) homogenate. The further dilution to have a 1:5 (*w*/*v*) homogenate was performed by adding proper volumes of HPLC-grade CHCl_3_ (chloroform). After vigorous vortexing, samples were centrifuged at 20,690× *g*, for 10 min at 4 °C; the clear supernatants were saved and pellets underwent a new homogenization step, using the same conditions described above. Samples were again vortexed and centrifuged and the resulting clear supernatants were combined with those saved after the first homogenization step. Mixing the two clear supernatants allowed us to obtain protein-free brain extracts (final tissue to liquid ratio = 1:10, *w*/*v*) in which the organic solvent was composed of 9 volumes of CH_3_CN and 1 volume of CHCl_3_. 

To determine the characteristics of the HPLC analysis, standard mixtures with increasing concentrations of reduced and oxidized CoQ_9_ and CoQ_10_ were submitted to the aforementioned extraction process and then analyzed to evaluate the sensitivity (as the lower limit of detection, LLOD, and lower limit of quantification, LLOQ) and linearity. Reproducibility was assessed by analyzing both the same standard mixture for five consecutive times on the same day (intra-assay variability) and five different standard mixtures (with same concentrations of the compounds of interest) on five consecutive days (inter-assay variability). To measure recovery, aliquots with low and high standard mixtures of reduced and oxidized CoQ_9_ and CoQ_10_ were added to CH_3_CN immediately before tissue homogenization. These spiked samples were then processed as described above, in order to determine whether the matrix (brain tissue) influenced the recovery of any of the reduced and oxidized CoQ_9_ and CoQ_10_.

The HPLC analysis was carried out using aliquots of 100 μL loaded onto a 150 × 4.6 mm, 5 µm particle size Hypersil Gold RP C-18 column provided with its own guard column (Thermo Fisher Scientific, Milan, Italy) and connected to a Surveyor HPLC system (ThermoFisher Italia, Rodano, Milan, Italy) equipped with a highly-sensitive photodiode array detector (provided by a 5 cm light path flow cell) and set up between 200 and 500 nm wavelength. Chromatographic separations of α-tocopherol, and reduced and oxidized CoQ_9_ and CoQ_10_, were carried out with 70% methanol + 30% H_2_O as the starting solvent A and 100% acetonitrile as the solvent B. A linear gradient from solvent A to solvent B was formed as follows: 0.5 min at 100% A; 8 min at up to 100% B (hold for additional 35 min). A flow rate of 1.0 mL/min and a column temperature of 37 °C were kept constant during the analysis. Data acquisition and analysis were performed using the ChromQuest^®^ software package (5.0 version) provided by the HPLC manufacturer. Calculations of the reduced and oxidized forms of CoQ_9_ and CoQ_10_ in unknown sample runs were performed at 290 or 275 nm wavelength, respectively, whilst that of α-tocopherol was carried out at 295 nm wavelength. Comparisons of retention times and area of peaks, with those of ultrapure standards with known concentrations, allowed exact quantifications of the compounds of interest.

### 2.4. Statistical Analysis

Statistical analysis was performed using the GraphPad Prism program, release 8.01 (GraphPad Software, San Diego, CA, USA). Normal distribution of the data was evaluated according to the Kolmogorov–Smirnov test. Differences among groups were determined by the 1-way analysis of variance for multiple comparisons, followed by the Tukey’s post-hoc test. Differences were considered significant when *p* < 0.05. Raw data of the CoQ values determined in control, mTBI and sTBI-injured rats have been added as supplementary material.

## 3. Results

### 3.1. Characteristics of the HPLC Method for the Simultaneous Determination of Reduced and Oxidized CoQ_9_ and CoQ_10_ in the Brain of Control Rats

Table 1 summarizes the parameters of sensitivity and linearity of the HPLC method. The lower limit of detection (LLOD) and the lower limit of quantification (LLOQ) of both reduced CoQ_9_ and CoQ_10_ were 10 (LLOD) and 15 (LLOQ) nM (corresponding to 1 and 1.5 pmol/100 μL injected, respectively) and that of both oxidized CoQ_9_ and CoQ_10_ were 20 (LLOD) and 30 (LLOQ) nm (corresponding to 2 and 3 pmol/100 μL injected, respectively). The method was highly linear, in a range of concentrations between LLOQ and 4000 × LLOQ. Since standard mixtures underwent the extraction procedure used to deproteinize the brain tissue, it is possible to affirm that these data strongly indicate no effects of the sample preparation process on the compounds of interest.

The reproducibility of the analytical HPLC method was assessed by determining intra-assay (five consecutive chromatographic runs of the same mixture) and inter-assay (five chromatographic runs of five, freshly prepared, standard mixtures analyzed on five consecutive days) coefficients of variations (CV) of peak areas and retention times (Table 2). The coefficients of the variations of peak areas and retention times were lower than 0.5% and 1.5% for retention times and peak areas in the case of intra-assay, and lower than 0.5% and 2% for retention times and peak areas in the case of inter assay. Even when performing these reproducibility tests, all standard mixtures underwent the same extraction process used for biological samples, prior to the HPLC analysis.

The recovery of the tissue processing of reduced and oxidized CoQ_9_ and CoQ_10_, as well as that of the HPLC method used for their determination, was evaluated by spiking acetonitrile (the organic solvent used for tissue protein removal and CoQs extraction) with either low (10 × LLOQ) or high (200 × LLOQ) reduced and oxidized CoQ_9_ and CoQ_10_ concentrations. These spiked acetonitrile solutions were then used to homogenize six brain hemispheres (three with 10 × LLOQ and three with 200 × LLOQ), according to the protocol described under Materials and Methods. Recovery, reported in Table 3 and Table 4, demonstrates a high efficiency of the method used for brain deproteinization and CoQ_9_ and CoQ_10_ extraction, either when acetonitrile was spiked with low (Table 3) or high (Table 4) concentrations of the compounds of interest.

**Figure 1:**
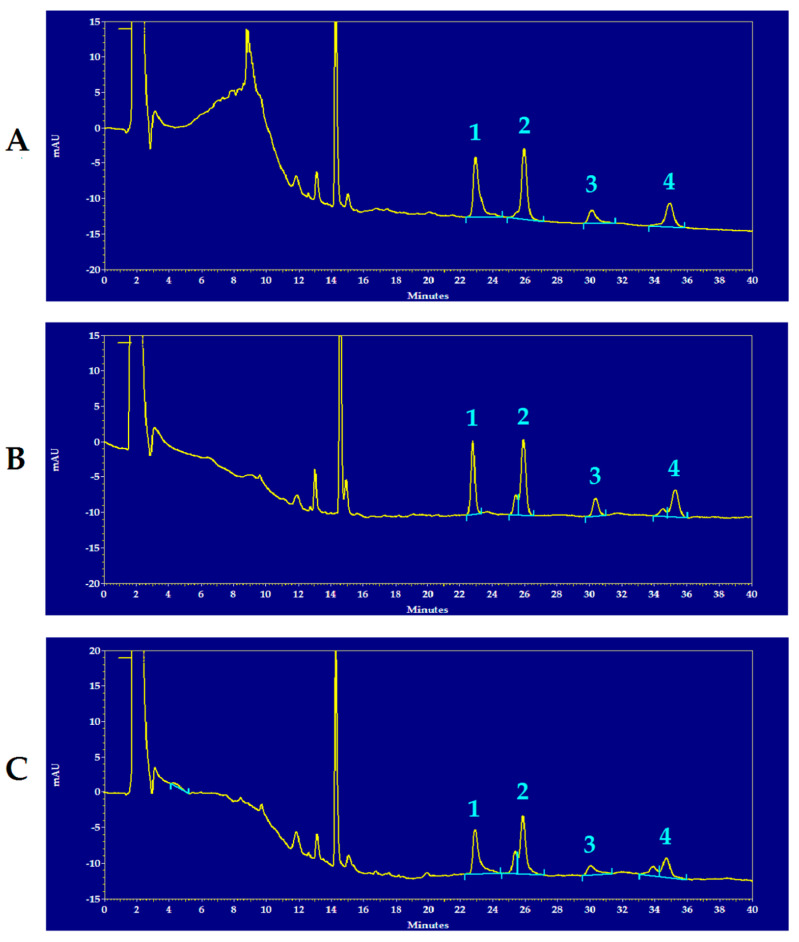
Representative chromatograms showing the HPLC separation of reduced and oxidized CoQ_9_ and CoQ_10_ in rat brain extracts of a control rat (**A**), an mTBI-injured rat (**B**) and an sTBI-injured rat (**C**). Numbered peaks correspond to 1 = reduced CoQ_9_; 2 = oxidized CoQ_9_; 3 = reduced CoQ_10_; 4 = oxidized CoQ_10_. The chromatographic runs are shown at 288 nm wavelength, in order to obtain easily visible peaks of the compounds of interest in one single run only. In each chromatogram, the peak at 14.4 min of retention time, corresponds to α-tocopherol. Full scale = 35 mAU, where AU = absorbance units.

Figure 1 shows representative chromatograms of the HPLC separation of reduced and oxidized CoQ_9_ and CoQ_10_ of the brain extracts of a control rat (**A**), of an mTBI-injured rat (**B**) and of an sTBI-injured rat (**C**), from which it is possible to observe the full separation of the compounds under evaluation, as well as visible differences in some of the peak areas of reduced and oxidized CoQ_9_ and CoQ_10_ in the chromatographic run of the sTBI animal (**C**).

### 3.2. Concentrations and Redox State of CoQ_9_ and CoQ_10_ in the Brain of Control Rats

**Figure 2:**
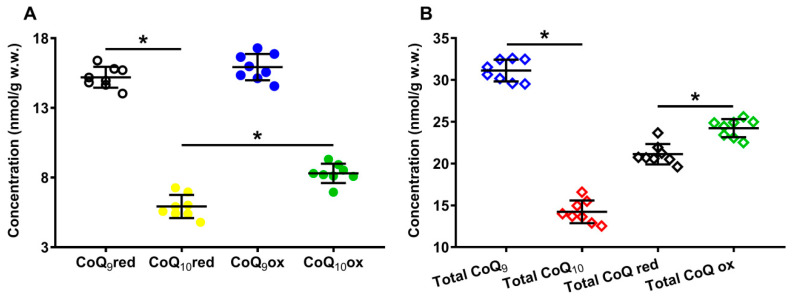
Concentrations of reduced and oxidized forms of CoQ_9_ and CoQ_10_ (**A**) detected in whole brain extracts of control rats. The total amounts (reduced + oxidized) of CoQ_9_ and CoQ_10_ and the total amounts of reduced (reduced CoQ_9_ + reduced CoQ_10_) and oxidized (oxidized CoQ_9_ + oxidized CoQ_10_) coenzymes Q forms (**B**) are also shown. Means, standard deviations and all data points (n = 8 control animals) are shown. CoQ_9_red = reduced coenzyme Q_9_; CoQ_10_red = reduced coenzyme Q_10_; CoQ_9_ox = oxidized coenzyme Q_9_; CoQ_10_ox = oxidized coenzyme Q_10_. * Significantly different, *p* < 0.001.

Panel **A** of Figure 2 shows the concentrations of the reduced and oxidized forms of CoQ_9_ and CoQ_10_ detectable, under physiologic conditions, in the organic solvent extracts of the whole brain of the control rats. In Panel **B** of the same Figure 2, the total amounts of CoQ_9_ and CoQ_10_, as well as the total reduced and oxidized coenzymes Q species, are indicated.

It is possible to observe that, under physiological conditions, while the rat brain has equal amounts of reduced and oxidized CoQ_9_ (15.19 ± 0.75 and 15.92 ± 0.94 nmol/g w.w., respectively), the concentration of reduced CoQ_10_ (5.92 ± 0.83 nmol/g w.w.) is 1.4 times lower than that of oxidized CoQ_10_ (8.30 ± 0.70, *p* < 0.001), suggesting the possible differential roles of CoQ_9_ and CoQ_10_ in the electron transport chain (ETC). 

**Figure 3:**
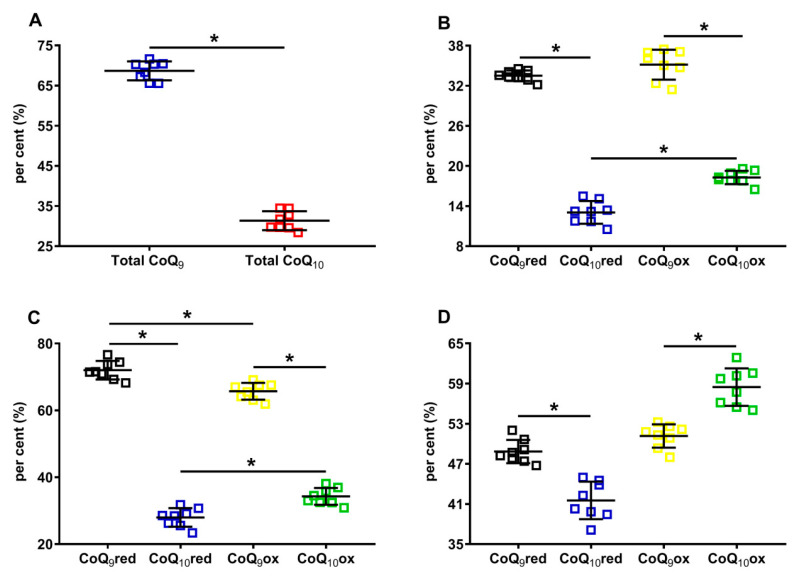
Per cent of cerebral CoQ_9_ and CoQ_10_, and of their respective reduced and oxidized species in brains of control rats. In (**A**), the per cent of total CoQ_9_ (reduced + oxidized) and total CoQ_10_ (reduced + oxidized) were calculated on the total coenzymes Q pool (reduced + oxidized CoQ_9_ + reduced + oxidized CoQ_10_). In (**B**), the per cent of reduced CoQ_9_ and CoQ_10_ and oxidized CoQ_9_ and CoQ_10_ were calculated on the total coenzymes Q pool (reduced + oxidized CoQ_9_ + reduced + oxidized CoQ_10_). In (**C**), the per cent of reduced CoQ_9_ and CoQ_10_ were calculated, respectively, on the amounts of total reduced coenzymes Q (reduced CoQ_9_ + CoQ_10_), whilst the per cent of oxidized CoQ_9_ and CoQ_10_ were calculated, respectively, on the amounts of total oxidized coenzymes Q (oxidized CoQ_9_ + CoQ_10_). In (**D**), the per cent of reduced and oxidized CoQ_9_ and reduced and oxidized CoQ_10_ were calculated, respectively, on the total CoQ_9_ (reduced + oxidized) or total CoQ_10_ (reduced + oxidized) levels. CoQ_9_red = reduced coenzyme Q_9_; CoQ_10_red = reduced coenzyme Q_10_; CoQ_9_ox = oxidized coenzyme Q_9_; CoQ_10_ox = oxidized coenzyme Q_10_. * Significantly different, *p* < 0.001.

Results separately considering the total concentrations of reduced + oxidized CoQ_9_ (31.12 ± 1.31 nmol/g w.w.) and CoQ_10_ (14.22 ± 1.36 nmol/g w.w., *p* < 0.001), show that the shorter form of coenzyme Q represents 68.86 ± 2.35% of the total coenzyme Q pool and CoQ_10_ only 31.14 ± 2.35% (Figure 3A). When dividing by the concentration of the total coenzyme Q pool, CoQ_9_ is distributed into equal amounts of reduced and oxidized CoQ_9_, whilst CoQ_10_ is characterized by a significantly higher per cent of the oxidized species (Figure 3B). 

When using the sum of reduced CoQ_9_ + CoQ_10_ or the oxidized CoQ_9_ + CoQ_10_ to calculate the different percentages, it was found that 72.03 ± 2.78% and 27.97 ± 2.78% corresponded to reduced CoQ_9_ or CoQ_10_, respectively, whilst 65.73 ± 2.51% and 34.27 ± 2.51% corresponded to oxidized CoQ_9_ or CoQ_10_, respectively. 

Lastly, when using the sum of reduced +oxidized CoQ_9_ and CoQ_10_ to calculate the different percentages, it was observed that CoQ_9_ was almost equally divided into reduced and oxidized (48.84 ± 1.74% and 51.16 ± 1.74%, respectively), whilst significantly lower values of reduced CoQ_10_ compared with oxidized CoQ_10_ were observed (41.53 ± 2.80% and 58.47 ± 2.80%, respectively, *p* < 0.0001). Overall, the different absolute values of CoQ_9_ and CoQ_10_, as well as the percent values of their respective reduced and oxidized forms, allowed for the hypothesis that rat brain mitochondria may have a different distribution within the three complexes of ETC using coenzymes Q as mobile electron transporters. 

**Figure 4:**
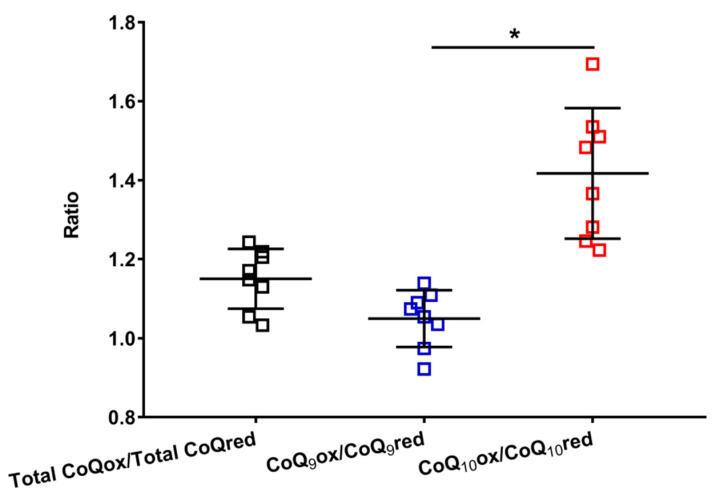
Values of the oxidized/reduced ratios of CoQ_9_ and CoQ_10_ found in whole brain extracts of control rats. Means, standard deviations and all data points (n = 8 control animals) are shown. Total CoQox = oxidized coenzyme Q_9_ + oxidized coenzyme Q_10_; Total CoQred = reduced coenzyme Q_9_ + reduced coenzyme Q_10_; CoQ_9_ox = oxidized coenzyme Q_9_; CoQ_9_red = reduced coenzyme Q_9_; CoQ_10_ox = oxidized coenzyme Q_10_; CoQ_10_red = reduced coenzyme Q_10_. * Significantly different, *p* < 0.001.

This hypothesis is supported by data reported in Figure 4, where the oxidized/reduced ratios of the total coenzymes Q, CoQ_9_ and CoQ_10_ are shown.

It is possible to observe that the total CoQox/total CoQred ratio is slightly higher than 1 (mean value = 1.15 ± 0.08), allowing us to suppose that more or less equimolar amounts of the oxidized and reduced species are stably present in the brain tissue, under physiological conditions. However, when separately considering the ratios of the short (oxidized CoQ_9_/reduced CoQ_9_) and long (oxidized CoQ_10_/reduced CoQ_10_) chained coenzymes Q, it was found that the former is equally distributed between the two oxidoreductive species (oxidized CoQ_9_/reduced CoQ_9_ mean value = 1.05 ± 0.07), whilst the latter is prevalently present in its oxidized species (oxidized CoQ_10_/reduced CoQ_10_ ratio mean value = 1.42 ± 0.17). This unequal distribution of CoQ_9_ and CoQ_10_ into the respective oxidized and reduced species seems to corroborate the possibility that the three complexes of ETC do not similarly use CoQ_9_ and CoQ_10_ in their respective oxidoreductive reactions.

### 3.3. Concentrations and Redox State of CoQ_9_ and CoQ_10_ in the Rat Brain following Graded TBI

**Figure 5:**
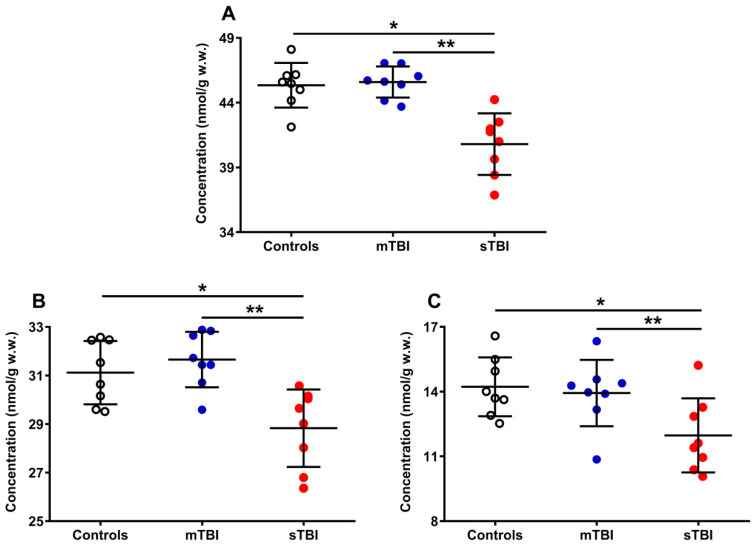
Concentrations of total CoQ pool (**A**), CoQ_9_ pool (**B**), and CoQ_10_ pool (**C**) detected in whole brain extracts of controls and rats sacrificed 7 days after experiencing mTBI or sTBI. Means, standard deviations and all data points (n = 8 in each group) are shown. Total coenzymes Q pool = reduced + oxidized CoQ_9_ + CoQ_10_; CoQ_9_ pool = reduced + oxidized CoQ_9_; CoQ_10_ pool = reduced + oxidized CoQ_10_. * Significantly different from controls, *p* < 0.001. ** Significantly different from mTBI, *p* < 0.001.

The results reported in Figure 5 show the differential effects of graded TBI (mild and severe) on the concentrations of the total pool of coenzymes Q (reduced + oxidized CoQ_9_ + CoQ_10_) (**A**), as well as on both the total CoQ_9_ (reduced + oxidized) (**B**) and CoQ_10_ (reduced + oxidized) (**C**) concentrations.

A week after mTBI, the concentration of the total CoQ pool (**A**) was not different from the value measured in the controls, whilst the amount of the total CoQ pool of sTBI rats (at the same time point post-impact) was significantly lower (−10%) than that measured in both controls and mTBI animals (*p* < 0.001). A similar situation was observed when separately considering the levels of total CoQ_9_ (reduced + oxidized) (**B**) and total CoQ_10_ (reduced + oxidized) (**C**). Animals experiencing mTBI had normal concentrations of both total CoQ_9_ and total CoQ_10_, whilst rats receiving sTBI showed a significant decrease in both short (CoQ_9_) and long (CoQ_10_) CoQ levels (*p* < 0.001 compared with both controls and mTBI). It is worth underlining that, when determining the per cent changes of the two coenzymes Q, it was found that a decrease in CoQ_9_ was −7.4% whilst that of CoQ_10_ was −18.6%, i.e., the per cent decrease in CoQ_10_ was 2.5 times more pronounced than that occurring in CoQ_9_.

**Figure 6:**
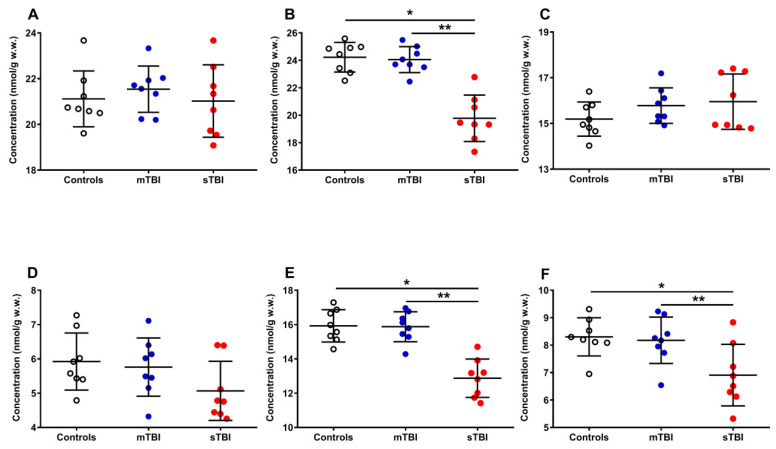
Concentrations of reduced CoQ_9_ + CoQ_10_ (**A**), oxidized CoQ_9_ + CoQ_10_ (**B**), reduced CoQ_9_ (**C**), reduced CoQ_10_ (**D**), oxidized CoQ_9_ (**E**) and oxidized CoQ_10_ (**F**) detected in whole brain extracts of controls and rats sacrificed 7 days after experiencing mTBI or sTBI. Means, standard deviations and all data points (n = 8 in each group) are shown. * Significantly different from controls, *p* < 0.001. ** Significantly different from mTBI, *p* < 0.001.

Figure 6 illustrates the effects of graded TBI on the total amount of reduced CoQ_9_ + CoQ_10_ (**A**) and oxidized CoQ_9_ + CoQ_10_ (**B**), as well as on the relative concentrations of reduced and oxidized species of both CoQ_9_ and CoQ_10_ (**C, D, E and F**), determined in the whole brain extracts of rats undergoing mTBI or sTBI. 

Whilst the amounts of reduced CoQ_9_ + CoQ_10_ (**A**) were not significantly modified by both mTBI and sTBI, the concentrations of oxidized CoQ_9_ + CoQ_10_ (−18.4%, **B**) underwent a remarkable decrease only in animals undergoing sTBI (*p* < 0.001, compared with both controls and mTBI rats). Both short and long reduced coenzymes Q were significantly affected neither by mild nor by severe TBI, although sTBI injured animals, compared with the controls (15.20 ± 0.75 and 5.92 ± 0.83 nmol/g w.w.), had slightly higher reduced (**C**) CoQ_9_ (15.96 ± 1.21 nmol/g w.w.) and lower reduced (**D**) CoQ_10_ values (5.07 ± 0.86 nmol/g w.w.). Oxidized CoQ_9_ (−19.5%, **E**) and CoQ_10_ (−16.9%, **F**) showed a significant decrease only when animals received sTBI (*p* < 0.001, compared with both controls and mTBI animals). 

**Figure 7:**
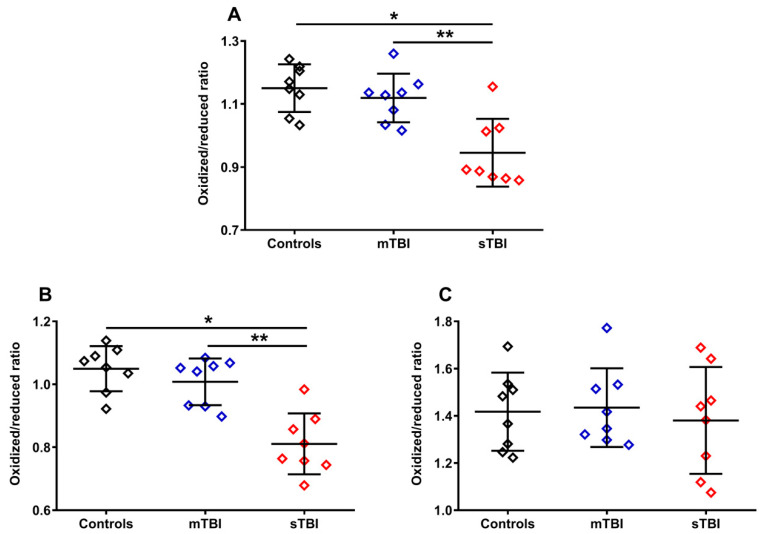
Ratios of oxidized CoQ_9_ + CoQ_10_/reduced CoQ_9_ + CoQ_10_ (**A**), oxidized CoQ_9_/reduced CoQ_9_ (**B**) and oxidized CoQ_10_/reduced CoQ_10_ (**C**) calculated from the values of the different forms (CoQ_9_ and CoQ_10_) and oxidoreductive species (reduced and oxidized) of coenzymes Q, determined in whole brain extracts of controls and rats sacrificed 7 days after experiencing mTBI or sTBI. Means, standard deviations and all data points (n = 8 in each group) are shown. * Significantly different from controls, *p* < 0.001. ** Significantly different from mTBI, *p* < 0.001.

To evaluate whether graded TBI differentially affected the oxidoreductive state of the two CoQ forms, we calculated the ratios of the oxidized/reduced species of the two CoQ forms (CoQ_9_ and CoQ_10_). As shown in Figure 7, the ratio of oxidized CoQ_9_ + oxidized CoQ_10_/reduced CoQ_9_ + reduced CoQ_10_ (**A**) had a −21.7% variation in animals undergoing sTBI (*p* < 0.001 compared with both controls and mTBI), due to the changes in the relative concentrations of the reduced and oxidized species of the two forms occurring only after a severe head injury (Figure 5A–F). Interestingly, when separately considering the oxidized/reduced CoQ_9_ and oxidized/reduced CoQ_10_ ratios, we found a −29.5% variation of the oxidized/reduced CoQ_9_ (**B**) only in sTBI-injured rats (*p* < 0.001 compared with both controls and mTBI) and no change (**C**) in the oxidized/reduced CoQ_10_. This suggests a potentially different location in the ETC of short and long chained CoQ and, moreover, potentially different functional effects of the changes in the oxidized/reduced ratios of the two forms (CoQ_9_ and CoQ_10_), on the overall mitochondrial energy-related activity.

**Figure 8:**
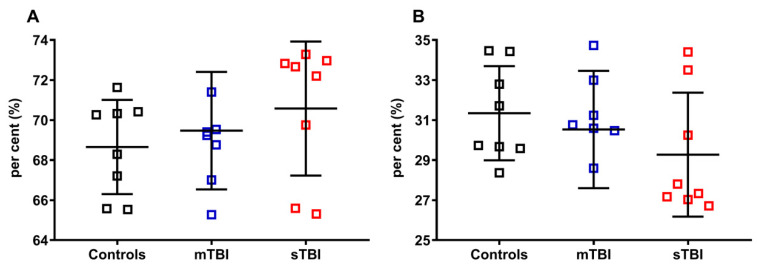
Per cent of total CoQ_9_ and total CoQ_10_ in brains of control rats and rats receiving graded TBI (mild mTBI, severe sTBI). In (**A**), per cent of total CoQ_9_ (reduced + oxidized CoQ_9_) was calculated on the total CoQ pool (reduced + oxidized CoQ_9_ + reduced + oxidized CoQ_10_). In (**B**), per cent of total CoQ_10_ (reduced + oxidized CoQ_10_) was calculated on the total CoQ pool (reduced + oxidized CoQ_9_ + reduced + oxidized CoQ_10_). Means, standard deviations and all data points (n = 8 in each group) are shown.

As indicated in Figure 8, neither mTBI nor sTBI influenced the per cent of total CoQ_9_ (reduced + oxidized CoQ_9_, **A**) and total CoQ_10_ (reduced + oxidized CoQ_10_, **B**), calculated on the total of the coenzymes Q pool (reduced + oxidized CoQ_9_ + reduced + oxidized CoQ_10_), although a visible tendency to increase the per cent of total CoQ_9_ (**A**) and decrease that of total CoQ_10_ (**B**) was observed in the case of sTBI animals. 

**Figure 9:**
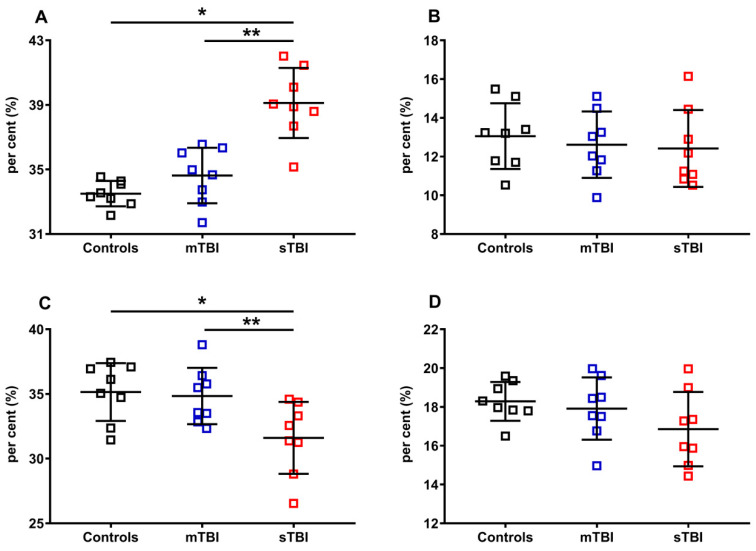
Per cent of reduced and oxidized CoQ_9_ and CoQ_10_ species in brains of controls rats and rats undergoing mild (mTBI) or severe (sTBI) traumatic brain injury. In (**A**), the per cent of reduced CoQ_9_. In (**B**), the per cent of reduced CoQ_10_. In (**C**), the per cent of oxidized CoQ_9_. In (**D**), the per cent of oxidized CoQ_10_. In all panels the per cent were calculated on the total CoQ pool (reduced + oxidized CoQ_9_ + reduced + oxidized CoQ_10_). Means, standard deviations and all data points (n = 8 in each group) are shown. * Significantly different from controls, *p* < 0.001. ** Significantly different from mTBI, *p* < 0.001.

Apparently, TBIs at both levels of severity did not influence the per cent of the reduced and oxidized species, when the reduced CoQ_9_ and CoQ_10_, or oxidized CoQ_9_ and CoQ_10_, were pooled and calculated on the total coenzymes Q concentration (reduced + oxidized CoQ_9_ + reduced + oxidized CoQ_10_). To verify this point, we separately considered reduced CoQ_9_ and CoQ_10_, or oxidized CoQ_9_ and CoQ_10_, then calculated the respective per cent using different dividers. In Figure 9, the per cent of the reduced and oxidized CoQ_9_ and CoQ_10_ were calculated on the total CoQ pool (reduced + oxidized CoQ_9_ + reduced + oxidized CoQ_10_).

Of the two forms of CoQ, only the per cent of the oxidoreductive species of the shorter chained CoQ (CoQ_9_) was affected by the highest level of injury tested (sTBI). A +16.8% of reduced CoQ_9_ and, consequently, an equal -16.8% of oxidized CoQ_9_ were calculated in rats receiving sTBI (*p* < 0.001), clearly indicating that graded TBIs differentially affect the oxidoreductive state of only one of the two CoQ forms.

**Figure 10:**
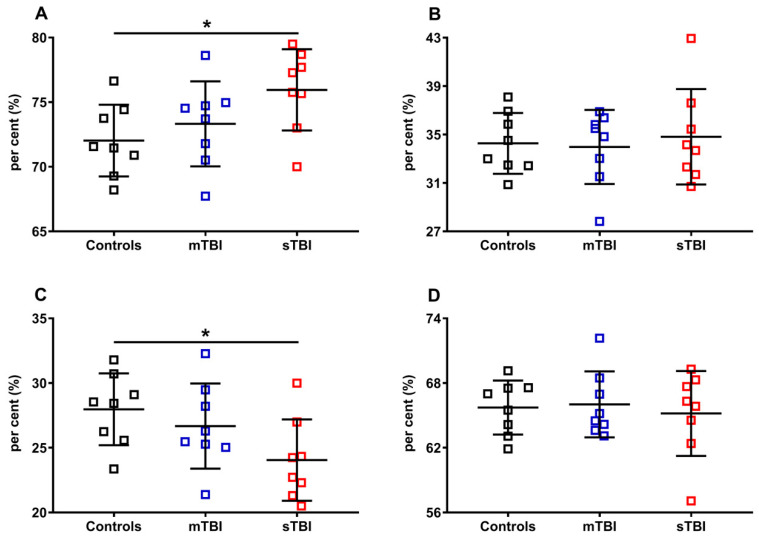
Per cent of reduced and oxidized CoQ_9_ and CoQ_10_ species in brains of controls rats and rats undergoing mild (mTBI) or severe (sTBI) traumatic brain injury. In (**A**), the per cent of reduced CoQ_9_. In (**B**), the per cent of reduced CoQ_10_. In (**C**), the per cent of oxidized CoQ_9_. In (**D**), the per cent of oxidized CoQ_10_. In (**A**,**B**), the per cent of reduced CoQ_9_ and CoQ_10_ were calculated on the total reduced CoQ species (reduced CoQ_9_ + reduced CoQ_10_). In (**C**,**D**) the per cent of oxidized CoQ_9_ and CoQ_10_ were calculated on the total oxidized CoQ species (oxidized CoQ_9_ + oxidized CoQ_10_). Means, standard deviations and all data points (n = 8 in each group) are shown. * Significantly different from controls, *p* < 0.001.

In Figure 10, the per cent of reduced and oxidized CoQ_9_ and CoQ_10_ were calculated, respectively, on the total of the reduced (reduced CoQ_9_ + reduced CoQ_10_) or oxidized forms (oxidized CoQ_9_ + oxidized CoQ_10_). Again, even when using this divider, we found that only the ratios of reduced and oxidized CoQ_9_ underwent significant changes, with a propensity to increase the amount of reduced CoQ_9_ and decrease that of oxidized CoQ_9_, when the brain was solicited by sTBI.

**Figure 11:**
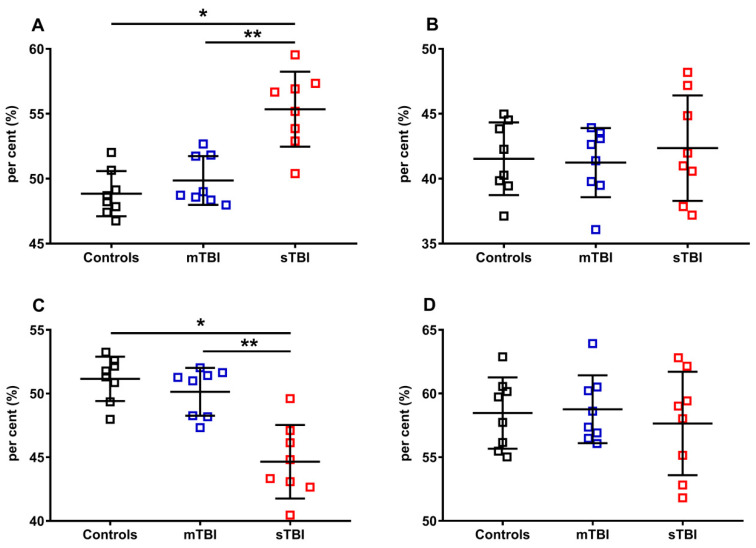
Per cent of reduced and oxidized CoQ_9_ and CoQ_10_ species in brains of controls rats and rats undergoing mild (mTBI) or severe (sTBI) traumatic brain injury. In (**A**), the per cent of reduced CoQ_9_, calculated on the total of CoQ_9_ (reduced + oxidized). In (**B**), the per cent of reduced CoQ_10_, calculated on the total of CoQ_10_ (reduced + oxidized). In (**C**), the per cent of oxidized CoQ_9_, calculated on the total of CoQ_9_ (reduced + oxidized). In (**D**), the per cent of oxidized CoQ_10_, calculated on the total of CoQ_10_ (reduced + oxidized). Means, standard deviations and all data points (n = 8 in each group) are shown. * Significantly different from controls, *p* < 0.05. ** Significantly different from mTBI, *p* < 0.05.

In order to fully evaluate the variations of the reduced and oxidized species of CoQ_9_ and CoQ_10_, we calculated their fluctuations in the controls, mTBI- and sTBI-injured rats, using as a divider the total amounts of CoQ_9_ (reduced + oxidized CoQ_9_) or CoQ_10_ (reduced + oxidized CoQ_10_). The results, illustrated in Figure 11, once again confirmed that only CoQ_9_ suffered from the effects of sTBI on the balance between the reduced and oxidized species (**A** and **C**). Indeed, at one week post-impact, sTBI animals had a −13.4% decline in the per cent of reduced CoQ_9_, paralleled by an equal +13.4% increase in the per cent of oxidized CoQ_9_ (*p* < 0.001 compared with both controls and mTBI). Overall, the calculations of the per cent changes of reduced and oxidized CoQ_9_ and CoQ_10_, performed using all the possible dividers, clearly showed that only the redox balance of the short-chained coenzyme Q is affected by sTBI, with a clear tendency to move the balance value towards the preponderance of the reduced CoQ_9_ species.

**Figure 12:**
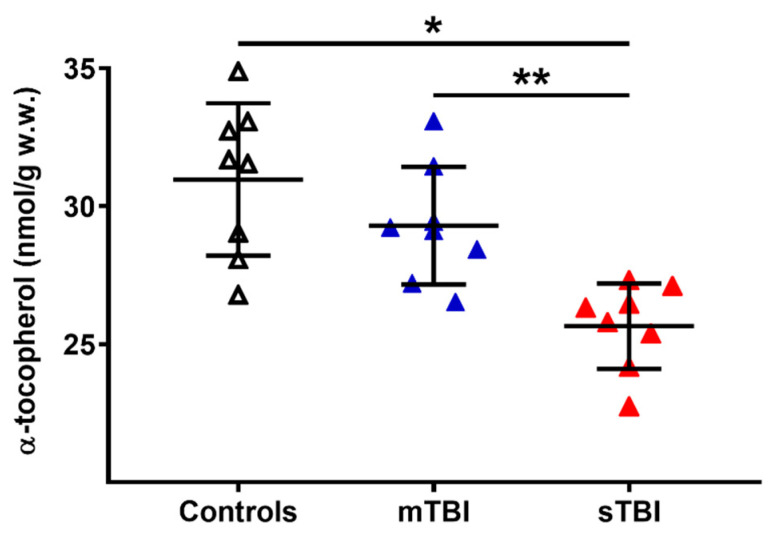
Concentrations of α-tocopherol in brains of controls rats and rats undergoing mild (mTBI) or severe (sTBI) traumatic brain injury. Means, standard deviations and all data points (n = 8 in each group) are shown. * Significantly different from controls, *p* < 0.01. ** Significantly different from mTBI, *p* < 0.01.

To evaluate a potential connection with the depletion of total CoQ levels following TBI, we also measured the cerebral concentrations of α-tocopherol, considered as the main fat-soluble antioxidant, the redox cycling of which is also performed with the intervention of reduced CoQ_9_ and CoQ_10_. As shown in Figure 12, whilst no changes occurred a week after mTBI, the levels of α-tocopherol in the brain tissue of sTBI rats at 7 days post-impact was −17% and −12% lower than the values measured, respectively, in the controls and mTBI animals (*p* < 0.01).

## 4. Discussion

The results of the present study, besides validating both the tissue processing for reduced and oxidized CoQ_9_ and CoQ_10_ and the HPLC method used for their determination, clearly evidenced the differential effects of graded TBIs on the concentrations and redox state of CoQ_9_ and CoQ_10_, as well as evidencing some potential peculiarities of the two shorter and longer chained CoQ present in cerebral rat tissue.

As reported in the literature, notwithstanding that CoQ_9_ represents almost the exclusive form of CoQ content (≅95–98% of total CoQ) in the majority of rat tissues (including those with high metabolic-rates such as liver, heart and muscle), the highest levels of CoQ_10_ are in the brain tissue. In accordance with previous studies [21,28], by applying a two-step extraction protocol followed by HPLC analysis, we confirmed that the total amount of CoQ in the rat brain is composed of 2/3 (about 65%) of CoQ_9_ and 1/3 (about 35%) of CoQ_10_. The main biochemical role of CoQ is to act as a mobile lipophilic electron carrier, collecting reducing equivalents from different sources: (i) NADH and FADH_2_ at the level of Complex I and II of the ETC, respectively; (ii) glycerol 3-phosphate dehydrogenase, one of the shuttle systems ensuring the entrance in the ETC of the cytoplasmic reducing equivalents generated by glycolysis; (iii) mitochondrial dihydroorotate dehydrogenase, a key enzyme involved in the pyrimidine biosynthetic pathway; (iv) electron transport flavoprotein dehydrogenases family (ETFDH), key enzymes of the fatty acid β-oxidation and branched-chain amino acid oxidation pathways [29,30]. Once reduced, due to its high hydrophobicity, CoQ travels within the inner mitochondrial membrane (IMM) and donates electrons to Complex III of the ETC, where it is involved in the well-known Q-cycle, a process terminating with the ratio between the reduced and oxidized states of CoQ in favor of the oxidized state in order to ensure the Q-cycle efficiency. Although potentially involved in various enzymatic functions, brain CoQ is almost completely devoted to guaranteeing the correct functioning of ETC, since glycerol 3-phosphate dehydrogenase, mitochondrial dihydroorotate dehydrogenase and ETFDH are poorly expressed in the brain tissue of rats. With this in mind, it is therefore possible that the differential fractional/percent levels of CoQ_9_ and CoQ_10_, identical to those of the ETC complexes donating electrons to CoQ (Complexes I and II = 2/3 of the ETC complexes using CoQ) and with that performing the Q cycle (Complex III = 1/3 of the ETC complexes using CoQ), are indicative of a higher affinity of Complexes I and II for CoQ_9_ and a higher affinity of Complex III for CoQ_10_, thus explaining the surprising coincidence indicated above. The reasons why the brain has the highest CoQ_10_ within the rat organism remain, however, unexplained.

Interestingly, when analyzing the redox states of the shorter and longer chained CoQ, we found that, although the total brain CoQ pool (CoQ_9_ + CoQ_10_) is composed of slightly equimolar quantities of reduced and oxidized forms, when considering CoQ_9_ and CoQ_10_ singularly, the shorter form is equally distributed in the reduced and oxidized state, whilst the reduced form of CoQ_10_ is 1.4 times lower than that of the oxidized form. It is reasonable to suppose that CoQ_9_, whose concentrations are equally distributed in the reduced and oxidized states, may be mainly associated with and involved in the collection of electrons from Complexes I and II, since it is highly probable that in properly working mitochondria equal amounts of the reduced and oxidized CoQ_9_ forms are needed for a continuous electron transfer to Complex III. Conversely, the 1.4 times higher levels of oxidized CoQ_10_ may be supportive of the hypothesis of the preferential use of the longer chained CoQ by Complex III, where at the end of a Q-cycle the oxidized/reduced CoQ ratio is increased. 

A second hypothesis regarding the different CoQ_9_ and CoQ_10_ levels and their redox states in the control rat brain tissue could be due to a dissimilar distribution of CoQ species among the so-called ETC supercomplexes. Recently, the “fluid model” of the ETC organization, in which protein complexes were viewed as independent entities embedded in the inner membrane, with CoQ and cytochrome c acting as mobile carriers that freely diffuse in the lipid membrane and inter-membrane space, respectively [31], has been replaced by the “plasticity” model characterized by the presence of, in addition to fixed and separated protein complexes, supermolecular protein assemblies, known as respiratory supercomplexes (SCs) [32,33]. SCs are functional quaternary structures that increase electron transport efficiency, reduce ROS production and stabilize the structure of free complexes, thus enhancing oxidative phosphorylation efficiency [34,35,36]. It has been found that, in mammalian mitochondria, Complex I is mostly associated with other complexes, either with a Complex III dimer (CI + CIII_2_) or with a Complex III dimer and a Complex IV monomer or dimer (CI + CIII_2_+CIV_1-2_, a superassembly known as N-respirasome) [37]. Complex III is usually found as a dimer (CIII_2_), but it can also create the SC known as Q-respirasome, in association with a Complex IV monomer or dimer (CIII_2_+CIV_1-2_) [38]. The co-existence of SCs and free complexes therefore suggests the possibility of a specific distribution of the CoQ pool in two functional populations and many scientific studies demonstrated the presence of a CoQ pool trapped within SCs containing a Complex I dedicated to transferring electrons originating from NADH (CoQ_NADH_) and a free CoQ pool diffused in the IMM utilized by Complex II and other FAD-containing enzymes (CoQ_FAD_) [39,40,41]. It may be hypothesized that the crucial role of mitochondrial cerebral energy metabolism and the peculiar phospholipid-rich composition of the brain tissue contribute to the use of CoQ_9_ in the electron transfer within SCs, and to cluster CoQ_10_, thanks to its higher hydrophobicity, in the shuttling of electrons between free complexes, providing a reasonable explanation for the co-existence of separate CoQ_9_ and CoQ_10_ pools in the rat brain, characterized by differential oxidized/reduced states ratio. 

In view of the key role of CoQ in ETC, but also in cellular antioxidant protection, alterations of CoQ homeostasis have been associated to pathological conditions characterized by mitochondrial dysfunction, such as neurodegenerative diseases [42,43,44]. Notwithstanding that TBI is characterized by energy impairment [45,46], mitochondrial malfunctioning [47,48] and sustained oxidative and nitrosative stress [49,50], nothing has been reported, to date, on possible changes in the levels and redox state of CoQ. Our results demonstrated, for the first time to the best of our knowledge, that TBI, depending on its severity, clearly affects either absolute concentrations or redox states of both CoQ_9_ and CoQ_10_ (although to different extents), adding new evidence to the overall mitochondrial impairment caused by TBI.

When evaluating the impact of TBI on CoQ homeostasis, the results of the present study clearly indicated that whilst mTBI rats had levels of total CoQ (reduced + oxidized CoQ_9_ + reduced + oxidized CoQ_10_), CoQ_9_ pool (reduced + oxidized) and CoQ_10_ pool (reduced + oxidized), as well as of those of reduced and oxidized CoQ_9_ and CoQ_10_, similar to those of the controls, sTBI animals had a significant depletion in total CoQ and in the pools of both shorter and longer chained CoQ levels. Additionally, the brain tissue of sTBI animals, a week after impact, showed differential alterations in the redox states of CoQ_9_ and CoQ_10_, with an overall selective decrease in the sum of oxidized CoQ species, but also differential alterations when separately considering the concentrations of reduced and oxidized CoQ_9_ and CoQ_10_ (Figure 5). Consequently, these unequal changes had different reflexes on the oxidized/reduced ratios of oxidized CoQ_9_ + CoQ_10_/reduced CoQ_9_ + CoQ_10_, oxidized CoQ_9_/reduced CoQ_9_ and oxidized CoQ_10_/reduced CoQ_10_, indicating that the decrease in the oxidized CoQ_9_ + CoQ_10_/reduced CoQ_9_ + CoQ_10_ ratio of the sTBI brain is solely due to the decrease in the ratio of oxidized CoQ_9_/reduced CoQ_9_ but not in that of the oxidized CoQ_10_/reduced CoQ_10_ (Figure 6).

Using the same rat model of graded diffused TBI, we previously showed that the mitochondrial quality control (MQC) system, finely regulating the mitochondrial dynamics through the processes of fusion, fission and mitophagy [51] and of crucial relevance for cell survival [52], undergoes selective changes depending on the severity of TBI. In particular, we found that, at 5 days post injury, while the mTBI-injured brain activated fusion and inhibited fission, thus promoting mitochondrial recovery, the sTBI-injured brain oppositely activated fission and mitophagy and inhibited fusion, with an overall decrease in the cerebral mitochondrial mass [16]. Therefore, it is conceivable that the profound alterations of MQC following TBI may contribute, through two possible mechanisms, to the depletion of the CoQ pool content occurring in the rat brain tissue following sTBIs only: (i) since sTBI induces sudden and long-lasting alterations in the mitochondrial phosphorylating capacity, causing energy crisis [45,46,53] and dysfunctional mitochondria [16,54], the brain tissue promptly activates the fission and mitophagy processes reducing the number of dysfunctional mitochondria consequently leading to an overall depletion of cerebral CoQ content; (ii) since sTBI induces a downregulation of the genes and protein expressions of mitofusins (MFN1 and MFN2 involved in the regulation of mitochondrial fusion), the decrease in MFN2, a component of the MQC involved in CoQ biosynthesis [29] and the maintenance of correct CoQ levels [55], may certainly contribute to the significant decrease in the CoQ content. 

**Figure 13:**
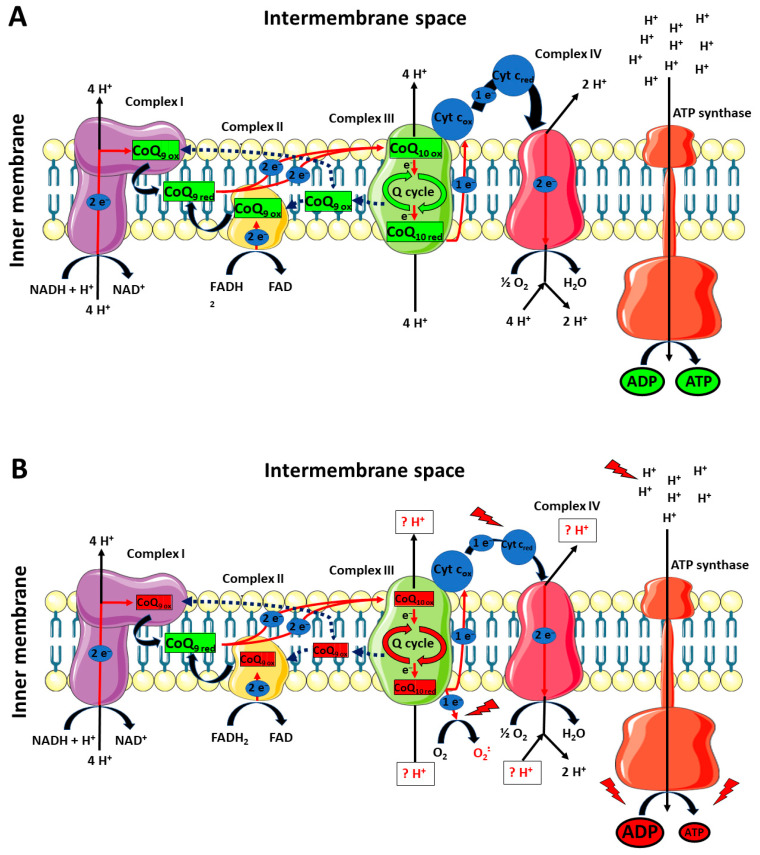
Schematic representation of the possible differential roles of CoQ_9_ and CoQ_10_ in rat brain mitochondria under physiologic conditions and a week after mTBI (**A**), or a week after sTBI (**B**). CoQ_9_ would preferentially be used by Complex I and II, ensuring the electron transfer to Complex III, where CoQ_10_ would be used to exploit the Q-cycle (**A**). A week after mTBI, no changes in the actual concentrations of reduced and oxidized CoQ_9_ and CoQ_10_ takes place. At 7 days post-sTBI (**B**), there is a significant decrease in both CoQ_9_ and CoQ_10_ levels. Additionally, change in the oxidized/reduced CoQ_9_ ratio is also observed, due to a slight increase in reduced CoQ_9_ and a decrease in oxidized CoQ_9_. This may be due to the slight decrease in the oxidized/reduced CoQ_10_ ratio, indicating an impairment in the Complex III-associated Q-cycle, indirectly responsible for the change in the oxidized/reduced CoQ_9_ ratio. The decreased efficiency of Complex III should create favorable conditions for the one-electron transfer to molecular oxygen, generating an overflow of superoxide anions. Furthermore, malfunctioning of the Q-cycle may cause defects in proton pumping in the intermembrane space by Complex III and Complex IV. In fact, diminished electron flow from Complex III to Complex IV, due to the electron leak when superoxide anions are formed, should even alter the efficiency in Complex IV proton translocation. The final result is an overall decreased mitochondrial capacity to phosphorylate ATP, with a subsequent cell energy crisis.

As previously mentioned, our results clearly showed that the impact of TBI was not only circumscribed to the decrease in cerebral CoQ_9_ and CoQ_10_ levels, but also to differentially modify their respective redox states. Once again, whilst no changes were found in mTBI animals at 7 days post-impact, sTBI-injured rats had significant imbalance in the oxidoreductive states of CoQ species. In particular, the concentrations of reduced CoQ_9_ + CoQ_10_ pool were not affected, whilst the concentrations of the oxidized pool underwent a remarkable decrease, thus inducing a significant decrease in the oxidized/reduced ratio of CoQ_9_ + CoQ_10_. When separately analyzing the concentrations of the four CoQ (reduced and oxidized CoQ_9_ and reduced and oxidized CoQ_10_) and the resulting oxidized/reduced ratios, it was observed that the oxidized/reduced ratio of CoQ_10_ was unaltered and that of CoQ_9_ underwent a significant decrease. This occurs as a consequence of a slight increase in reduced CoQ_9_ and a decrease in oxidized CoQ_9_, accompanied by an equal decrease in reduced and oxidized CoQ_10_. Therefore, compared with the values of the control rats, the resulting values of the respective ratios clearly indicates a more reduced state of CoQ_9_ and no change in that of CoQ_10_. These findings suggest that the differential redox imbalance of CoQ_9_ and CoQ_10_ may be due to an unequal impairment of the ETC complexes. In fact, the significant tendency to shift the balance value towards the preponderance of the reduced CoQ_9_ species, besides corroborating the hypothesis that CoQ_9_ is mainly used by Complexes I and II and CoQ_10_ by Complex III, may mainly be due to the decreased efficiency of the Q cycle (occurring in Complex III), causing an overall slowdown of the electron flux through ETC, a progressive accumulation of the reduced CoQ_9_ species, a decreased efficiency in proton pumping in the mitochondrial inner membrane space and a consequent impairment in OXPHOS-dependent ATP production. A schematic representation of these hypotheses is illustrated in Figure 13 (Panels A and B).

Taken together, these hypotheses are supported by previous studies reporting an overall downregulation of ETC complexes, particularly affecting Complex IV, in different animal models of TBI [53,56,57]. Using the same TBI animal model, we previously demonstrated the occurrence of permanent downregulations of pyruvate dehydrogenase (PDH) and selective tricarboxylic acid cycle enzymes [15], an increase in lactate production and a decrease in the NAD^+^/NADH ratio in sTBI animals only [14]. The present results may help to hypothesize that such phenomena take place through a sort of feedback mechanism induced by the decreased efficiency of ETC and the consequent accumulation of reducing equivalents in the mitochondrial membrane (decrease in the oxidized/reduced ratio of CoQ_9_) and matrix (decrease in the NAD^+^/NADH ratio), acting as negative modulators of PDH activity and expression [58].

In addition to the effect on CoQ homeostasis, the present study also showed that sTBI only provokes a significant depletion of the cerebral concentration of α-tocopherol, confirming the occurrence of decreased antioxidant defenses with consequent oxidative stress following severe head injury [3,45,49,59]. According to our interpretation of a potential clustering of CoQ_10_ as the preferential CoQ form to ensure efficiency of the Q-Cycle at the Complex III level, and keeping in mind that the oxidized/reduced ratio of CoQ_10_ was slightly affected by sTBI suggesting the potentially altered activity of Complex III, the decrease in α-tocopherol may just be due to the incorrect exploitation of the Q-cycle, during an overall malfunctioning of ETC, using molecular oxygen for the oxidation of the semiquinone radical, generating ROS overflow and decreasing α-tocopherol content.

## 5. Conclusions

To the best of our knowledge, the results reported in the present study highlighted, for the first time, TBI’s effects on the brain’s CoQ homeostasis, causing not only a significant decrease in the brain CoQ pool, but also an imbalance of the two oxidoreductive species occurring in the rat brain. Specifically, the CoQ homeostasis appears to suffer profound alterations following sTBI only. Combined with previous findings obtained using the same TBI animal model [14,15,16,17,45,47,49], it is now possible to conclude that, following mTBI, all metabolic pathways and cycles, independently from their cytoplasmic, mitochondrial matrix or mitochondrial membrane localizations, completely recover their functionality after a week post injury, ensuring a mitochondrial capacity to satisfy the cerebral cells energy demand. Conversely, it is now possible to add that, after sTBI, as a metabolic feature of the sTBI-post-injured brain, there is a long-lasting, possibly irreversible, CoQ depletion and an alteration of its redox state, both of which have profound influences on mitochondrial dysfunction and the consequent energy crisis. Therefore, we here provided the rationale for the development of new pharmacological treatments aimed to restore CoQ homeostasis and ETC functionality. In this light, due to the plethora of biochemical, metabolic and molecular alterations induced by TBI to brain cells, multi-drug administration protocols might represent the choice of election to ameliorate TBI patients’ treatment. It is evident that increased efforts should be dedicated to elucidating the molecular mechanisms, inducing alterations of the ETC functionality and connecting CoQ alterations to those occurring in specific ETC complexes, that fully characterize the complex TBI-mediated biochemical/metabolic/molecular changes of cerebral cells.

## Figures and Tables

**Table 1:** Lower limit of detection, lower limit of quantification and linearity of the reversed phase HPLC method for the detection of reduced and oxidized CoQ_9_ and CoQ_10_.

Compound	Retention Factor *k’*	LLOD nM	LLOQ nM	4000 × LLOQ μM	Correlation Coefficients of Linearity Straight Lines
Reduced CoQ_9_	13.32	10	15	60	0.999
Oxidized CoQ_9_	15.13	20	30	120	0.997
Reduced CoQ_10_	17.38	10	15	60	0.998
Oxidized CoQ_10_	20.61	20	30	120	0.999

*k’* = V−V_0_/V_0_, where V = the elution volume of the compound considered and V_0_ = the void volume of the chromatographic system. LLOD = lower limit of detection, evaluated with a signal to noise ratio > 3. LLOQ = lower limit of quantification, evaluated with a signal to noise ratio > 10. Linearity was determined by assaying standard mixtures of reduced and oxidized CoQ_9_ and CoQ_10_ with the following concentrations: LLOQ, 10 × LLOQ, 20 × LLOQ, 50 × LLOQ, 500 × LLOQ, 1500 × LLOQ and 4000 × LLOQ.

**Table 2:** Reproducibility of the HPLC method for the separation and detection of reduced and oxidized CoQ_9_ and CoQ_10_.

Compound	Intra-Assay Coefficient of Variation of Retention Times	Intra-Assay Coefficient of Variation of Peak Areas	Inter-Assay Coefficient of Variation of Retention Times	Inter-Assay Coefficient of Variation of Peak Areas
Reduced CoQ_9_	0.16 ± 0.04	0.85 ± 0.12	0.25 ± 0.07	1.28 ± 0.25
Reduced CoQ_10_	0.19 ± 0.06	1.01 ± 0.17	0.36 ± 0.09	1.64 ± 0.19
Oxidized CoQ_9_	0.18 ± 0.05	0.77 ± 0.08	0.28 ± 0.03	1.48 ± 0.30
Oxidized CoQ_10_	0.20 ± 0.03	0.54 ± 0.06	0.33 ± 0.06	1.51 ± 0.14

Values are the mean ± SD of five consecutive chromatographic runs of the same standard mixture, in the case of the intra-assay variability, whilst they are the mean ± SD of five chromatographic runs of five different standard mixtures assayed in five consecutive days, in the case of the inter-assay variability.

**Table 3:** Recovery of the reversed phase HPLC method for the detection of reduced and oxidized CoQ_9_ and CoQ_10_ in brain tissue extracts.

Compound	Mean Values in Control Brain Samples (µmol/L Brain Extract)	Concentration Added = 10 × LLOQ (µmol/L)	Expected Mean Values (µmol/L Brain Extract)	Mean Measured Values (µmol/L Brain Extract)	Mean Recovery (%)	%SDR
Reduced CoQ_9_	1.49 ± 0.06	0.15	1.64	1.59 ± 0.05	97.0	3.1
Reduced CoQ_10_	0.61 ± 0.03	0.15	0.76	0.73 ± 0.07	96.1	9.6
Oxidized CoQ_9_	1.75 ± 0.07	0.30	2.05	1.98 ± 0.11	96.6	5.6
Oxidized CoQ_10_	0.87 ± 0.06	0.30	1.17	1.22 ± 0.07	104.3	5.4

Each value is the mean ± SD of three different brain extract samples. Three brain extracts of control rats were extracted and analyzed with no addition, in order to determine the basal values of the compounds of interest. Acetonitrile was spiked, immediately before its use to deproteinize brain tissue samples, with a standard mixture at low concentration (10 × LLOQ) of reduced and oxidized CoQ_9_ and CoQ_10_. Sample processing and chromatographic conditions are fully described in the Materials and Methods section.

**Table 4:** Recovery of the reversed phase HPLC method for the detection of reduced and oxidized CoQ_9_ and CoQ_10_ in brain tissue extracts.

Compound	Mean Values in Control Brain Samples (µmol/L Brain Extract)	Concentration Added = 200 × LLOQ (µmol/L)	Expected Mean Values (µmol/L Brain Extract)	Mean Measured Values (µmol/L Brain Extract)	Mean Recovery (%)	%SDR
Reduced CoQ_9_	1.46 ± 0.14	3	4.46	4.59 ± 0.19	103.0	4.1
Reduced CoQ_10_	0.54 ± 0.08	3	3.54	3.46 ± 0.20	97.7	5.8
Oxidized CoQ_9_	1.78 ± 0.12	6	7.78	7.48 ± 0.16	96.1	2.1
Oxidized CoQ_10_	0.84 ± 0.06	6	6.84	7.05 ± 0.14	103.1	2.0

Each value is the mean ± SD of three different brain extract samples. Three brain extracts of control rats were extracted and analyzed with no addition, in order to determine the basal values of the compounds of interest. Acetonitrile was spiked, immediately before its use to deproteinize brain tissue samples, with a standard mixture at high concentration (200 × LLOQ) of reduced and oxidized CoQ_9_ and CoQ_10_. Sample processing and chromatographic conditions are fully described in the Materials and Methods section.

## Data Availability

Raw data are available as an excel file in Supplementary Materials.

## Supplementary Materials

The following supporting information can be downloaded at: https://www.mdpi.com/article/10.3390/antiox12050985/s1.
